# Performance Comparison between Densified and Undensified Silica Fume in Ultra-High Performance Fiber-Reinforced Concrete

**DOI:** 10.3390/ma13173901

**Published:** 2020-09-03

**Authors:** Sung-Hoon Kang, Sung-Gul Hong, Juhyuk Moon

**Affiliations:** 1 Department of Architecture and Architectural Engineering, Seoul National University, 1 Gwanak-ro, Gwanak-gu, Seoul 08826, Korea; medesis@snu.ac.kr; 2 Department of Civil and Environmental Engineering, Seoul National University, 1 Gwanak-ro, Gwanak-gu, Seoul 08826, Korea; 3 Institute of Construction and Environmental Engineering, Seoul National University, 1 Gwanak-ro, Gwanak-gu, Seoul 08826, Korea

**Keywords:** ultra-high performance fiber-reinforced concrete, densified silica fume, agglomeration, pozzolanic reaction, densification

## Abstract

Silica fume (SF) is a key ingredient in the production of ultra-high performance fiber-reinforced concrete (UHPFRC). The use of undensified SF may have an advantage in the dispersion efficiency inside cement-based materials, but it also carries a practical burden such as high material costs and fine dust generation in the workplace. This study reports that a high strength of 200 MPa can be achieved by using densified SF in UHPFRC with Portland limestone cement. Additionally, it was experimentally confirmed that there was no difference between densified and undensified SFs in terms of workability, compressive and flexural tensile strengths, and hydration reaction of the concrete, regardless of heat treatment, because of a unique mix proportion as well as mixing method for dispersing agglomerated SF particles. It was experimentally validated that the densified SF can be used for both precast and field casting UHPFRCs with economic and practical benefits and without negative effects on the material performance of the UHPFRC.

## 1. Introduction

Ultra-high performance fiber-reinforced concrete (UHPFRC) is a construction material that has excellent mechanical properties, durability, and flowability. Its development was attributed to the achievement of optimal packing density by the use of ultrafine particles such as silica fume (SF) and quartz powder, the prevention of brittle failure by the inclusion of the high volume of short fibers, and advances in the technology of chemical admixtures [1,2]. The demand for this commercial material has increased in earnest since the beginning of this century, mainly related to innovative and sustainable structures such as building facades, infrastructures, and protection or explosion-proof facilities [3,4]. In these circumstances, practical factors such as price competitiveness, sustainability, accessibility of raw materials, and worker safety are becoming increasingly important concerns. With regard to the sustainability of this material, one major challenge was to reduce its enormous content of Portland cement (up to 1000 kg/m^3^) or unnecessary unhydrated cement [5,6]. Therefore, the incorporation of various supplementary cementitious materials (SCMs) such as limestone powder, calcined clay, fly ash, ground granulated blast furnace slag, steel slag, and metakaolin has been attempted in order to replace a portion of the cement [7,8,9,10,11,12,13]. Considering the practical aspect in particularly, cement replacement by limestone powder has been regarded as one of the most effective solutions [14,15,16,17,18,19].

To enhance UHPFRC’s market competitiveness, it is also important to reasonably determine the type and content of silica fume (SF), considering economic efficiency and usability. SF is one of the most widely used ultrafine particles today, but, at the same time, it is also one of the most expensive construction materials due to its limited supply [20]. Therefore, inevitably, the more the material is incorporated, the higher the material cost of concrete [21]. After pioneering studies on the optimization of packing density by the use of ultrafine particles [1,2,22], it has been generally agreed that the optimal ratio of SF to the weight of cement is 25% in UHPFRC [6,23]. However, this ratio is at least two to three times higher than other types of concrete [20], and this gap is even more overwhelming when compared based on the content per unit volume of concrete [24].

Although SF makes a significant contribution to UHPFRC’s material price, the use of alternative materials must be approached cautiously because SF is one of the most important raw materials affecting almost all material properties of UHPFRC. The ball bearing effect, which acts as a lubricant by the combination of SF and superplasticizer, and the micro filling effect that fills the space between fine particles or refines the capillary pore structure contribute decisively to exhibiting the unique characteristics and superior performance of UHPFRC [25]. The yield stress of fresh UHPFRC is reduced by the ball bearing effect, which guarantees its superior self-compacting ability [23]. The micro filling effect makes cement paste extremely compact and increases the interfacial bond between aggregates and the paste [26,27]. SF is also responsible for making the fiber matrix interface area; as an example, the interfacial bond strength within the steel fiber matrix is significantly affected by the content of the ultrafine particles [28]. SF particles that can be adsorbed on a cement particle up to millions also affect the chemical reaction of UHPFRC [29]. By providing a site for nucleation of calcium silicate hydrate (C-S-H), it accelerates cement hydration and directly participates in secondary hydration (i.e., a pozzolanic reaction) [30,31]. In particular, the latter not only reduces the content of portlandite, which negatively affects the strength and durability of the cement paste, but it also forms additional C-S-H, which improves these properties [32]. For these reasons, although it has been previously reported that SCMs composed of amorphous silica can replace the chemical roles of SF [33], it has been suggested that, with these materials, it is difficult to completely replace all the complicated roles of the SF in UHPFRC, especially in terms of the physical effects carried out by spherical particles with submicron size [9,24,25]. This further supports the extreme difficulty in finding materials that can replace SF in UHPFRC.

As another alternative, the use of densified SF can be considered, and this can be a practical compromise as it has been the type of product commonly used in the concrete industry [29,34]. When collected in a silo as a produced form, SF originally has a density of 125 to 150 kg/m^3^ [35]. However, an additional process for densification of ultrafine particles proceeds due to the improvement of convenience in handling and transportation (also related to material cost) and workers’ health problems caused by fine dust (also related to labor cost) [36]. When air is blown into the silo, the particles roll and agglomerate in tens of μm to several mm, so that its density increases in the range of 480 to 720 kg/m^3^ [37]. Although undensified SF with a density of less than 350 kg/m^3^ is also used in limited amounts for special applications such as those involving pre-bagged products and grouts [38], it is not a widely used form for concrete production due to its high price, caused by low demand and the inconvenience involved with transportation and storage, as well as dust generation in the workplace [37]. However, despite disadvantages in usability and price competitiveness, undensified products have been preferred as suitable SFs in the production of UHPFRC because they can guarantee better dispersion than a densified product. Indeed, improved homogeneity compared to other types of concrete was one of the basic principles in the development of UHPFRC, and it has been considered that homogeneous dispersion of ultrafine particles is a prerequisite prior to particle size and specific surface area (SSA) of ultrafine particles [1]. For this reason, despite the practically great advantages, the feasibility of the densified product and its effect on the material properties of UHPFRC have been barely investigated.

When studying concrete with SF, its agglomeration tendency should be taken into account since SF particles do not exist in the form of independent nanoparticles but almost always as lumps [34,39]. This situation has been commonly reported in concrete containing coarse aggregate despite partial crushing of the lumps during the mixing process [40,41]. The agglomeration phenomenon is no exception in the SF manufactured as a collected state [42]. In other words, undensified SF can also not be free from agglomeration issues, so it is difficult to guarantee its complete dispersion within UHPFRC [43]. Moreover, due to the nature of the agglomeration, it is also considered very challenging to accurately measure the particle size or size distribution of SF [41]. Although ultrasonic dispersion or sonification before measurement is somewhat effective [44], it did not show completely satisfactory results because it could not affect the strong electrical attraction of the whole particles [41,45,46,47]. In general, laser diffraction (LD) and dynamic light scattering (DLS) are used for particle size analysis of powder materials, with advantages such as short analysis time and good reproducibility [48,49]. These techniques are suitable for particles with sizes between 0.5–3500 μm and 0.5–3 to 5 μm, respectively [49,50]. In this context, it has been suggested that it is reasonable to exclude the results of >1 μm range that are clearly larger than the size of separated SF particles while including the ultrasonic dispersion process [34,35]. Similarly, DLS techniques can be considered to characterize silica nanoparticles as they can naturally exclude large fractions of agglomerates depending on the measurement range of the device [25,32,51].

The main purpose of this study is to investigate the feasibility of using densified SF in order to improve the usability and price competitiveness of UHPFRC.

## 2. Materials and Methods

Three types of SF products with different manufacturers, manufacturing methods, and ages were prepared and then their effects on the material properties of the concrete were experimentally examined. First of all, to characterize SF products, chemical analysis by X-ray fluorescence (XRF), morphological analysis by high-resolution field emission scanning electron microscopes (FE-SEM), individual particle size calculation by the image processing technique, particle size distribution measurement by DLS, and SSA measurement by the BET method were carried out. Additionally, the performance of UHPFRC with different SF products was evaluated by measuring slump spread and mechanical properties such as compressive and flexural tensile strengths. Finally, X-ray diffraction (XRD) and thermogravimetric (TG) analyses were performed to compare the hydration mechanism due to the different form of SF on the properties of the concrete.

### 2.1. Preparation of UHPFRC Samples

**Figure 1:**
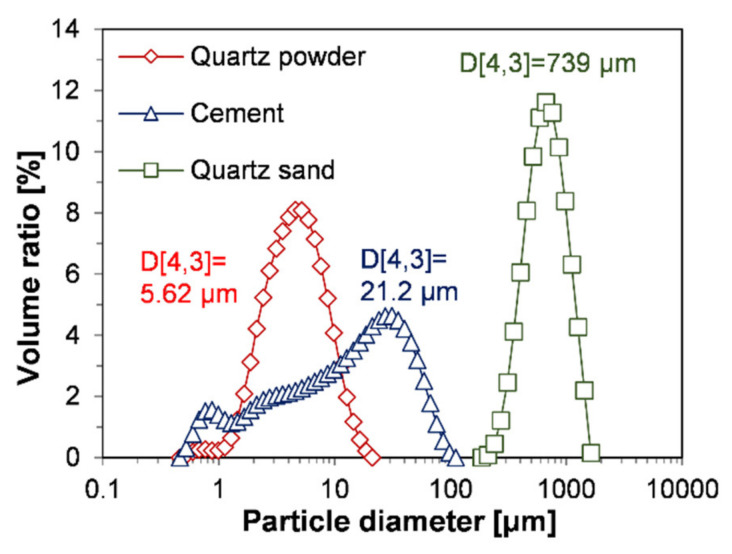
Particle size distribution of raw materials by laser diffraction.

For the experiment, UHPFRC was manufactured using Portland limestone cement, SF, quartz powder (SiO_2_ > 97 wt %), quartz sand (SiO_2_ > 90 wt %), water, polycarboxylate (PCE)-based superplasticizer, and steel fiber (Φ0.2 mm × 13 mm, tensile strength > 2500 MPa), as in previous studies [16,17]. The mineralogical composition of the used cement is monoclinic alite (46.79 wt %), triclinic alite (6.94 wt %), monoclinic belite (2.19 wt %), orthorhombic belite (1.38 wt %), anhydrite (0.61 wt %), aluminate (0.88 wt %), gypsum (1.09 wt %), dolomite (12.36 wt %), calcite (21.38 wt %), and amorphous content (6.38 wt %) measured by quantitative XRD analysis. The results of particle size analysis of the raw materials by LD are shown in Figure 1. Considering the measurement range of the equipment used (Mastersizer 3000, Malvern Panalytical, UK), the size distributions of cement, quartz powder, and quartz sand were measured. The size of quartz powder was in the range of 1 to 20 μm; moreover, its sizes were located within the range of the particle size of cement (0.4 to 100 μm), confirming that the quartz powder can properly perform its primary role in UHPFRC, i.e., filling the space between cement particles [1].

Three different SF products (labeled as SF1_U, SF2_U, and SF2_D) were prepared as experimental variables. SF1_U is a high quality, undensified SF product available worldwide. It is named Microsilica-Grade 940U (Elkem, Oslo, Norway) and its bulk density is 200 to 350 kg/m^3^ according to the manufacturer’s specifications. Around three years had passed since the purchase date of the product, but when estimated based on the date of manufacture, a much longer period should have elapsed considering import, distribution, and storage by domestic dealers. SF2_U and SF2_D are undensified and densified SF products manufactured in the same factory (POSCO, Pohang, Korea) and have bulk densities of ~180 and ~500 kg/m^3^, respectively. These domestic products, which do not require shipping or long-term transport and storage, had not been in our possession more than one year from the date of manufacture. As shown in Table 1, analysis results of chemical composition by a wavelength dispersive XRF spectrometer (S4 PIONEER, Bruker, Germany) verify that all products met the purity requirement of SF (>96 wt %) suitable for UHPFRC [23]. One reason that purity is important is that the inclusion of impurities, especially unburned carbon, has serious adverse effects on the hydration, mechanical properties, and workability of UHPFRC [24]. The results of XRF analysis also confirm that all SF products have the ability to function as highly reactive pozzolans and that their chemical compositions can be excluded from experimental parameters.

**Figure 2:**
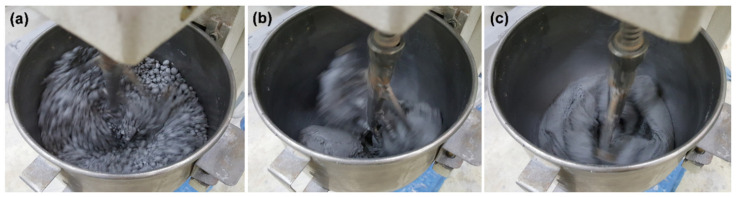
Manufacturing process of UHPFRC: (**a**) formation of small grains by local agglomeration; (**b**) agglomeration of small grains into large lumps; (**c**) dispersion after solid-suspension transition.

UHPFRC samples were prepared based on the mix proportions shown in Table 2; this was done with a 5-L planetary mixer. SF was blended with quartz sand at low speed (140 rpm) for 5 min to break up the lumped, ultrafine particles by collision with hard grains. This is possible because the collisions between particles enable dispersive movement, which causes collapse and dispersion of the agglomerated particles [52]. The remaining powders (cement and quartz powder) were then blended for another 5 min after being placed into the mixer. After 10 min of the dry blending process, water and superplasticizer were poured into the mixer and low speed mixing was maintained until the mixtures were agglomerated. As shown in Figure 2, the agglomeration of the mixture proceeded in two stages, during which local agglomeration of the relatively small grains was followed by the formation of one large agglomerate due to the agglomeration of the small grains.

When mixing is maintained for a certain period of time after the liquid materials are added, a fluid bond is formed between the particles so that the materials in the mixer begin to agglomerate, as shown in Figure 2a. The interparticle force between particles increases due to the surface tension of the water and the capillary pressure inside the bond, which requires the mixer to increase its power to further disperse the particles; eventually, dispersion efficiency increases [53]. When all the ingredients in the mixer were agglomerated, the power of the mixer reached its maximum, as shown in Figure 2b. Immediately after this, a transition occurred between the solid aggregate and the dispersed suspension; at this time, the particles inside the aggregate were evenly distributed and the space between the particles was filled with liquid. Once this transition occurred, the capillary force disappeared so that the power consumption of the mixer was drastically reduced. Consequently, the mixture began to have self-compacting abilities, as shown in Figure 2c. After the steel fibers were added into the mixture, which had been changed to the suspension state, they were mixed at a high speed (285 rpm) for 2 min as the last step of the manufacturing process.

The freshly prepared UHPFRC was filled in the prepared mold. The concrete was poured slowly at one end of the mold when casting prismatic specimens to exclude the effect of fiber orientation. All specimens were cured in a sealed state at 20 °C on the first day. The next day, heat treatment was applied. In other words, the specimens were cured at 90 °C for 2 days after demolding, as the standard heat treatment method. From the third day to the experimental day, specimens were cured in a chamber set at 20 °C and 60% relative humidity (RH). In addition, the samples without heat treatment were also prepared. In this case, the specimens were exposed to air drying conditions (20 °C and RH 60%) from the next day after casting.

### 2.2. Experimental Methods

For morphological analysis of raw materials, microscopic observation was performed using FE-SEM equipment (JSM-7800F Prime, JEOL Ltd., Tokyo, Japan). Powder samples were fixed on a carbon tape bonded holder and the upper surface of the holder was coated with carbon to prevent the charging effect. The holder was then inserted into the microscope and images of the particles were captured at a magnification of up to ×100,000. Additionally, the SEM images of SF were used to obtain its size distribution based on individual spherical particles. Since the results can significantly vary depending on the number of particles or images, the image processing technique was performed according to the method specified in ISO 13322-1 [54].

As another method for obtaining the particle size distribution of SF, an analysis using DLS was performed. To improve dispersion efficiency, the sample was sonicated for 5 min in an ultrasonic bath (53 kHz and 200 W) and then the size distribution was measured at a set temperature (25 °C) by the device (Zetasizer Nano ZSP, Malvern Panalytical, UK). Moreover, SSA of SF samples was determined by the BET method, for which the nitrogen adsorption desorption isotherms were recorded using a surface area analyzer (Autosorb IQ-MP/XR, Quantachrome Instrument, Boynton Beach, FL, USA).

To measure the material properties of UHPFRC, a series of experiments was conducted on workability, compressive strength, and flexural tensile strength. Workability of the fresh concrete was evaluated by the use of a flow table test without shock [55]. Compressive strength tests were performed according to ASTM C109 [56]; on the 28th day, cube specimens with one side of 50 mm were loaded by a universal testing machine and the average of the three specimens was determined as the strength value. On the same day, flexural tensile strength was measured by the three-point bending test method specified in ISO 679 [57]. For this, line loads were applied to three prismatic specimens (40 mm × 40 mm × 160 mm) by the machine.

XRD and TG analysis were performed to investigate the effect of SF on the hydration reaction of UHPFRC. To improve the precision of the analysis results, paste samples (excluding the materials that do not contribute to the chemical reaction, such as fine aggregate and steel fibers) were additionally prepared on the same day as the UHPFRC samples. On the 28th day, the hydrated paste was crushed and ground into a powder. Thereafter, the hydration reaction of the samples was stopped using isopropanol and diethyl-ether, according to the solvent exchange method [58,59]. After the pretreatment, the powders were placed on sample holders and then mounted on an X ray diffractometer (SmartLab SE, Rigaku, Tokyo, Japan). XRD patterns were collected by Cu·Kα_1_ radiation (λ = 1.5406 Å) under the established conditions, such as voltage: 40 kV; current: 40 mA; step size: 0.02°, and scanning speed: 1°/min. The patterns collected between 5° and 70° were analyzed by SmartLab Studio II software (Rigaku, Tokyo, Japan), equipped with an NIST inorganic crystal structure database and crystallography open database.

TG analysis was performed to quantitatively estimate the consumption of portlandite due to the pozzolanic reaction. Around 50 mg of powder samples was weighed in an alumina made sample holder which was placed on an analytical instrument (SDT 650, TA Instruments, New Castle, DE, USA). Under an environment where nitrogen gas was introduced at the rate of 100 mL/min, the samples were heated from 30 to 1000 °C at the rate of 10 °C/min. The weight loss with increasing temperature was recorded and the derivative thermogravimetric (DTG) was presented in the graph along with the weight loss to clearly identify the sudden weight change in a specific temperature range.

## 3. Results and Discussion

### 3.1. Observation of Agglomerated SF Particles

**Figure 3:**
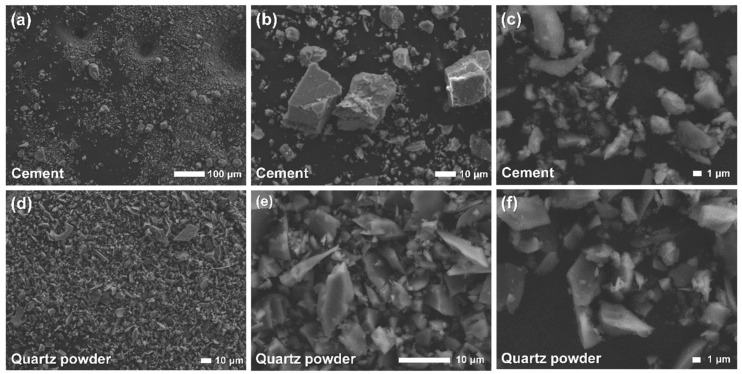
SEM images of cement (**a**–**c**) and quartz powder (**d**–**f**) with various resolutions.

Figure 3 shows SEM images of cement and quartz powders at various magnifications. As depicted below, these μm sized particles do not aggregate together but exist separately. The cement is composed of particles with a wide range of sizes from ~1 to ~50 μm (Figure 3a–c). In the case of quartz powder, the large particles were around 20 to 30 μm and the small ones were around several μm (Figure 3d–f). Compared to the cement, quartz powder showed a narrow particle size range because the crushed rock was sieved within a specific size range during its manufacture. The observation of particle size by SEM tended to be consistent with the result of LD-based analysis, as shown in Figure 1. This implies that particle size analysis for completely separated powders can ensure good reliability.

**Figure 4:**
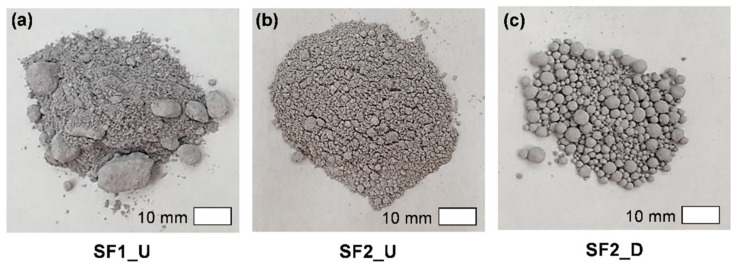
Visual observation of SF samples as a state used for manufacture of UHPFRC: (**a**) SF1_U, (**b**) SF2_U, and (**c**) SF2_D.

Prior to SEM observation of the SF particles, visual inspection was performed as shown in Figure 4. Although the sample SF1_U is an undensified product, many lumps large enough to match the size of the coarse aggregates were observed. This was probably due to the agglomeration during a long period of shipment, transportation, and storage. Moreover, during this period, a vertical load was applied by the other products stacked on top of it. On the other hand, such large lumps were not found in the sample SF2_U, which should have been less affected by the factors related to aggregation after manufacturing. As expected, the sample SF2_D consisted of globular lumps due to the densification process during manufacturing. The size of the lumps was up to several mm, and, unlike irregular ones in the sample SF1_U, they were almost perfectly spherical. This means that, once lumps are formed during the densification process, they are difficult to agglomerate thereafter, and thus this type of product has advantages for quality control.

**Figure 5:**
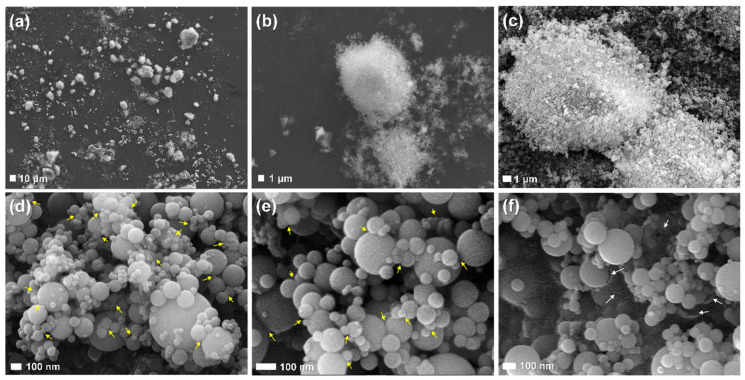
SEM images of SF1_U samples with various resolutions: agglomerated lumps (**a**–**c**) and spherical nanoparticles (**d**–**f**) inside lumps.

The true nature of these lumps can be viewed in detail by the use of SEM images (Figure 5). Figure 5a shows dust-like lumps that were observed. As mentioned in the introduction, the spherical nanoparticles never existed independently. The size of the lumps was various, and large ones of several tens of μm were also seen. The magnified images are presented in Figure 5b,c, in which myriad nanoparticles were agglomerated together to form the lumps. It has been reported that such lumps can consist of up to several tens of millions of nanoparticles [41]. As shown in Figure 5d–f, where individual nanoparticles are observed, it was confirmed that their size is tens to hundreds of nm based on separated spheres. In particular, some particles are not separate spheres but rather they appear to melt and stick together (yellow arrows in Figure 5d,e). Another interesting discovery was that of broken particles (white arrows in Figure 5f), indicating that the SF particles also have the shape of a hollow sphere, like other spherical SCMs such as fly ash.

It is generally known that SF particles are mostly (>95%) composed of nanoparticles [27]. However, it should be noted that a significant part of the SF particles has a form of linked spheres, regardless of the densification process. Spherical nanoparticles that are connected as a result of sintering or fusion have been previously observed [39]. As also found in this study (Figure 5d,e), consequently, general forms of SF particles are aggregates of several spheres rather than independent spheres [37]. This type of connection is completely different from the agglomeration due to the densification process because it is a very strong and irreversible bond. When high purity quartz is converted to silicon at a high temperature (~2000 °C), SiO_2_ vapor is generated; the vapor oxidizes and condenses at a low temperature while forming spherical particles with a size of 100 to 200 nm, but when they come into contact with each other in the molten state, primary aggregation occurs [60]. Due to this, dozens to hundreds of spheres are connected, and, typically, the size of these clusters has been reported to be 500 to 800 nm [41]. Such clusters formed by primary aggregation cannot be separated unless they are broken. For this reason, there has been confusion about the effective size of SF in all cases: immediately after manufacture, at the point of use, and after being incorporated into concrete [35].

**Figure 6:**
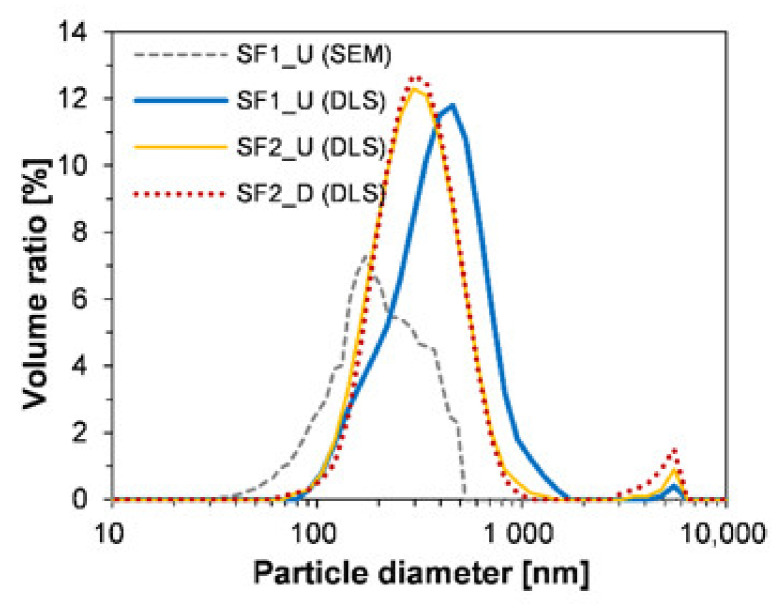
Particle size distribution of SF samples by DLS and SEM-based image processing techniques.

Figure 6 shows the particle size distribution of SF samples by DLS and image processing techniques. By comparing the results of SF2_U (DLS) and SF2_D (DLS), the effectiveness of the technique (DLS with ultrasonic treatment)—which excludes the effect of the densification process—can be verified. As evident from visual observation (Figure 4), there was a significant difference in the degree of aggregation between the two samples, but, nevertheless, there was almost no difference in particle size distribution, as shown in Figure 6. In both samples, the main peak was formed between 70 nm and 1 μm. However, in the size of the peak formed between 1 and 10 μm, the densified sample was larger than the undensified sample due to the contribution of large, aggregated particles. For this reason, SF2_D showed a higher value than SF2_U in the average particle size, as shown in Table 3. Meanwhile, by comparing the two yellow and blue curves, differences can be observed between the two products classified by the manufacturer. Regarding the proportion of the particles with sizes of >400 nm, the SF1_U sample had a higher value than SF2_U and SF2_D, which also resulted in a difference in average particle size (Table 3). This is presumably due to an increase in the agglomeration of small particles during the manufacturing process or thereafter. Moreover, the agglomeration phenomenon that had become stronger over a long period of time could have lowered the dispersion efficiency by the ultrasonic treatment.

Outcomes of the difference between the image processing and DLS techniques can be confirmed by comparing the two results of SF1_U (SEM) and SF1_U (DLS) (see gray dotted line and blue solid line in Figure 6). As expected, compared to DLS, the image processing technique formed the particle size distribution in a small size range, since it did not reflect the agglomeration phenomenon at all. Both methods have their own limitations. In particular, DLS can overestimate the particle size of SF because it measures hydrodynamic size (not a static or dry particle size) [61]. Nevertheless, this technique can be considered more suitable than the SEM-based image processing technique to measure the size distribution of SF particles. This is because effective particle sizes have more significant meanings than those that are independent due to their agglomerating nature [22]. Furthermore, the use of a high-resolution microscope is time consuming and expensive, and only a limited number of particles included in the 2D image are considered in the results. Indeed, results from previous studies have suggested that the actual size observed by SEM is unable to reflect the realistic particle size distribution because SF is always present in a clustered or agglomerated state [25,41].

The SSAs of the samples are shown in Table 3. The measured value was 22.9 to 25.3 m^2^/g and the deviation between the minimum and maximum was around 10%. This is consistent with the results of the previous study in that SSA of SF was in the order of 20 m^2^/g, despite diversity in type or manufacturer [41]. Moreover, there was no significant difference among the three samples of SSA, which is one of the most important parameters for the chemical effect of SCM on cement-based materials [32,45]. Unlike other natural or industrial byproduct-based SCMs, one of the advantages of SF is very low quality variation in terms of chemical composition, particle size, and SSA, which can vary by factory and manufacturer [62]. In this aspect, the measurement results in this study showed this benefit well.

### 3.2. Material Properties of UHPFRC with Various Types of Silica Fume

The measured flow diameters were 270, 260, and 255 mm for the fresh UHPFRC samples containing SF1_U, SF2_U, and SF2_D, respectively. According to the international standard on UHPFRC, their workability class is Cv, meaning viscous UHPFRC with self-compacting ability [63]. When comparing the two UHPFRC samples containing SF2_U and SF2_D, there was almost no difference, although the flow diameter was slightly higher than when undensified SF was used. If other conditions are the same, as the SF is more evenly dispersed in UHPFRC, the yield stress is further reduced due to the ball bearing effect [23], thereby increasing the self-compacting ability [52]. This implies the possibility that there was no significant difference in the degree of dispersion of SF particles inside the two fresh concrete samples. Meanwhile, between the two samples containing undensified SFs, when SF2_U was used, the flow diameter was 10 mm lower than the result of SF1_U. Regarding the decrease in flowability, both the dispersion efficiency of SF and the difference in SSA can be complicated because, as the SSA increases, water demand and friction between the particles increase [48]. Nonetheless, overall, there was no significant difference in workability among the samples, suggesting that there was no significant difference in the SSA and the dispersion efficiency of SF in the concrete samples.

**Figure 7:**
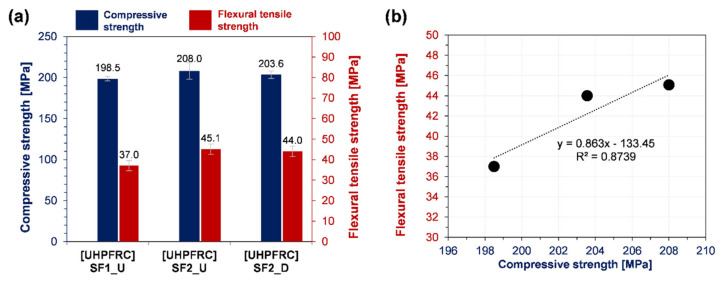
Results of strength tests: (**a**) compressive and flexural tensile strengths; (**b**) the relationship between the two strengths.

The experimental results of compressive strength and flexural tensile strength are presented in Figure 7a. Above all, every sample exhibited a very high compressive strength of around 200 MPa. Compared to the sample [UHPFRC], SF2_U showed the highest strength. [UHPFRC] SF1_U was ~10 MPa lower but [UHPFRC] SF2_D was ~5 MPa lower. This is an important result for the feasibility of using densified SF in UHPFRC because it confirms the negative effects that can be caused by the densification process of SF, which are negligibly small in the mechanical properties of UHPFRC. In addition, the concrete’s excellent strength can be ensured by using this commercially optimized product. Based on the results of [UHPFRC] SF2_U, the difference in concrete strength with the same type of product from another company ([UHPFRC] SF1_U) was more pronounced than with the other type of product from the same company ([UHPFRC] SF2_D). Various material properties affect SF concrete performance, including chemical composition, SSA, particle size or size distribution, the amount of silanol groups (Si-OH) on the particle surface, and post production age [64]. This suggests the diversity of properties related to SF1_U and SF2_U which affect the performance of UHPFRC. On the other hand, in terms of the difference in such properties of SF2_U and SF2_D, factors other than the densification process can be ignored. For this reason, the feasibility of using densified SF in UHPFRC can be proposed. Results from the flexural tensile strength test further support this suggestion. When compared to 45.1 MPa of the sample [UHPFRC] SF2_U, the strength of sample [UHPFRC] SF2_D was only 1.1 MPa (or 2.4%) lower. However, the strength of the sample [UHPFRC] SF1_U was 8.1 MPa (18%) lower than this. The flexural strength results were consistent with those of compressive strength, as confirmed by the clear linear correlation in Figure 7b. In summary, the parameters such as the densification process and the manufacturer or storage period affected the compressive strength and the flexural tensile strength with the same tendency. More importantly, the former parameter had little effect on both compressive and tensile properties of UHPFRC.

Direct measurement of the dispersion degree of SF in cement-based materials is extremely challenging [34]. Thus, indirectly, mechanical properties can be considered as an indicator for evaluating this. To date, the benefits expected from the use of undensified SF (such as improvement in dispersion efficiency in concrete and thereby improvement in mechanical properties) have been reported. This can be explained by enhancements in the role of SF such as the micropore filling effect, pozzolanic reaction, and the provision of nucleation sites. However, comparisons between different types of SF products have been mainly investigated in cement composites other than UHPFRC, such as the concrete and mortar types that contain coarse aggregates or have relatively high *w*/*c* [48,65]. Moreover, results from previous studies confirmed that a large amount of SF lumps (>10 μm) existed in such cement composites [34,39]. In practical situations, the only way to disperse SF products inside concrete or mortar is with mechanical crushing by a mixer, which is also one of the most effective methods [66]. When mixing concrete, crushing and shearing actions are transferred to the lumps of SF particles by a mixer and the dispersion efficiency is highly dependent on the mixture composition and mixing method. However, it has been consistently reported that densified SF cannot be satisfactorily dispersed in the cement composites, which is completely different from UHPFRC in terms of the mixing method and procedure [66,67,68,69].

When UHPFRC is manufactured, SF particles should be effectively dispersed by a suitable mixing method and a superplasticizer; otherwise, fundamental principles such as the optimization of mixture composition or packing density are meaningless and, in the end, the excellent performance of the concrete cannot be guaranteed [23]. The manufacturing process of this concrete includes one unique state in which all the ingredients in the mixer are agglomerated like flour dough (Figure 2b). At this point, when the mixer’s power consumption reaches its maximum, a strong shearing action is applied to the agglomerate so that the components, including the SF particles, can be effectively dispersed. Moreover, in the presence of steel fibers, additional dispersion is possible in the subsequent high-speed mixing process. Although the perfect dispersion of individual SF particles might be impossible [39], the lumps formed by the densification process are weakly connected and thus can be easily crushed by agitation. Therefore, UHPFRC’s unique formulation and manufacturing process can be effective in removing reversible aggregation by the densification process. As a result, there may not be a significant difference between densified and undensified products in dispersion efficiency in this concrete.

Along with compressive strength and durability, excellent crack resistance performance is one of the main features of UHPFRC, which can contribute to the construction of innovative and sustainable concrete structures [70]. This is the reason that the strength under flexural or tensile loading is an important property of this concrete [71]. Regarding this, one notable result was that, unlike the compressive strength, which increased due to the promoted pozzolanic reaction, there was no noticeable change in flexural tensile strength despite the increase in temperature (60 to 90 °C) and duration (up to 4 days) during heat treatment [72]. This is because the properties of steel fibers (aspect ratio, shape, surface treatment, etc.) and the distribution state of the fibers inside the concrete (volume ratio, direction, degree of dispersion, etc.) have a decisive effect on flexural or tensile properties [73,74,75,76], but the difference between these factors could be neglected in this study. Apart from such promotion of the pozzolanic reaction and the change in fiber parameters, the effect of the mixture composition on tensile strength has also been reported. In the study by Chan and Chu, the interfacial bond strength of the fibers increased proportionally with the contents of undensified SF (between 0 and 30 wt % by cement), which was attributed to the improvement in friction and resistance force of the fibers as the interface became denser [28]. In addition to these previous results, those from this study revealed that the type of SF also affects the tensile properties of UHPFRC, and more importantly, there is no significant reduction in the strength despite the use of densified SF.

### 3.3. Hydration Reaction of Heat-Treated UHPFRC with Various Types of Silica Fume

**Figure 8:**
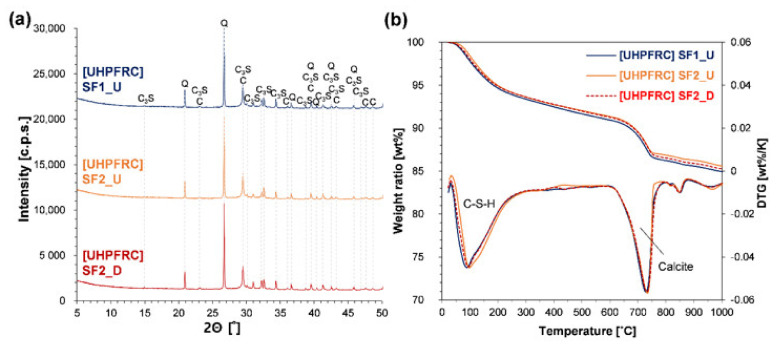
Analysis of crystal phases and hydration products of heat-treated paste samples: (**a**) XRD patterns at 28 days (Q: quartz, C: calcite, C_3_S: tricalcium silicate); (**b**) TG and DTG curves at 28 days.

Figure 8a,b show the results of XRD and TG analysis of heat-treated paste samples at 28 days. Typical phases identified in the XRD pattern of this concrete are quartz, calcite, cement clinkers, ettringite, and portlandite, but the last two phases were not detected in this study. The absence of ettringite at 28 days confirms that it was decomposed at an elevated temperature (>70 °C), and delayed ettringite formation did not occur during the subsequent 25 days of curing [77]. In particular, the cause of the latter is the extremely compact microstructure and low *w*/*c* of UHPFRC; the effect of heat treatment makes this condition even more dramatic because elevated temperature curing (or heat treatment) significantly promotes both cement hydration and pozzolanic reaction. These further fill capillary pores while consuming a limited amount of water [78]. However, despite the heat treatment, unhydrated cement clinkers still existed (as shown in Figure 8a). This is the result of an insufficient amount of water to fully hydrate the cement and the lack of space for further forming hydration products [79,80]. Additionally, these conditions do not provide water and space for the reformation of ettringite [81], and, because of its excellent watertightness, external water cannot penetrate into the concrete, even under water curing conditions [80]. The weight loss and DTG curves shown in Figure 8b clearly confirm that ettringite was not formed after decomposition. This is because sudden weight loss at around 105 °C is associated with dehydration of ettringite, and a sharp peak on the DTG curve due to this loss could not be observed [58]. Therefore, the gentle peak formed between 40 and 300 °C can only be attributed to the dehydration of C-S-H.

Another notable result from XRD and TG analysis is the absence of portlandite. The presence of portlandite was clearly confirmed in the previous study [72], in which all conditions other than the cement (i.e., raw materials, mixing method, and curing program) were the same as with this study. Additionally, in other studies, although the pozzolanic reaction was significantly promoted by the heat treatment at 90 °C, portlandite was not completely consumed [31,82,83]. In the study by Korpa et al., which includes in-depth discussions of the phase development of UHPFRC conditions based on XRD and TG analyses, the presence of portlandite and hence the ongoing progress of the pozzolanic reaction after 28 days was reported [84]. In UHPFRC, this crystal has been reported to be completely consumed by the pozzolanic reaction that was promoted by the heat treatment at >200 °C [85]. Unlike previous studies mentioned, the cement used in this study contained a significant amount of limestone powder, which was likely to contribute to the complete consumption of portlandite. The cause can be explained as follows: substitution of limestone powder instead of cement at a given amount of water increases the effective *w*/*c* (0.3 in this study). Additionally, since portlandite is produced by the primary cement hydration, a decrease in the content of primary hydration products due to the cement dilution effect can deplete portlandite even when the amount of accessible water is increased. The previous result also supports this, in that the portlandite content in UHPFRC decreased proportionally with the increase in the content of limestone powder [17].

However, even when a significant portion (up to 74%) of cement was replaced with limestone powder, portlandite could not be completely consumed without heat treatment [15,17]. This is because the reactivity of SF at room temperature is significantly low, and thus, even if a small amount of portlandite is formed, the role of SF related to consumption of this crystal is bound to be limited. The activation energy of SF required to participate in the pozzolanic reaction has been reported to be approximately 80 kJ/mol [86]. In this regard, heat treatment can greatly accelerate the reactivity of SF. It has also been reported that the solubility of amorphous silica increases proportionally with the temperature of water or solution [87]. Pfeifer et al. noted that the reaction degree of SF in UHPFRC was around 5% under 28 days of the ambient curing condition (20 °C), but the degree rapidly increased to around 45% (around nine times) when the standard heat treatment was applied to the concrete [31]. They also reported that an increase in effective *w*/*c* significantly improves the proportion of SF participating in the chemical reaction (around three times when *w*/*c* increases from 0.2 to 0.4). For this reason, even under the conditions in which a small amount of portlandite is formed due to the use of limestone powder, complete consumption of portlandite in UHPFRC would not be possible without heat treatment.

In addition to reducing the content of the cement, the use of limestone powder in UHPFRC has several advantages; autogenous shrinkage is alleviated by the increase in effective *w*/*c* [17,18] and the initial hydration is accelerated due to the provision of nucleation site for the formation of C-S-H [16]. Furthermore, the complete consumption of portlandite found in this study can provide additional advantages regarding the use of Portland limestone cement in UHPFRC. The pozzolanic reaction by SF or the consumption of portlandite has been known to persist for several years [88], and even after heat treatment, the compressive strength of UHPFRC increases continuously for 6 to 8 years due to this chemical reaction [89]. Indeed, an inversely linear relationship between the compressive strength and portlandite content has been reported in UHPFRC. This can be explained by the formation of additional C-S-H and refinement of the pore structure and removal of portlandite which has a morphologically undesirable effect on the strength of concrete and hardness of the interfacial transition zone [72]. Moreover, in terms of durability, this crystal can cause an expansion reaction with ions penetrated from the outside, thereby leading to cracking. Consequently, since the pozzolanic reaction has a decisive influence on the long-term strength and durability of UHPFRC [24], the complete consumption of portlandite shown herein suggests that the maximum mechanical properties and durability of this concrete can be guaranteed within 28 days. The stabilization of long-term properties is certainly an advantage when considering only practical usability because it can provide a reliable design strength for informing the work of practitioners and structural engineers [23].

In addition to the reactivity of SF, the chemical composition, particle size distribution, SSA, and degree of dispersion are all important factors in the chemical reaction of concrete [37,90]. In this regard, the results in Figure 8, in which no portlandite was detected in any of the samples, confirm that there might be no difference between densified and undensified SF products in the hydration and pozzolanic reaction of UHPFRC. This also implies that, between them, there was no difference in the degree of dispersion. This can be more evident if the same result is confirmed under the situation in which portlandite is not fully consumed.

### 3.4. Feasibility of Using Densified Silica Fume in Field Casting UHPFRC

An important application type of UHPFRC is field casting concrete (e.g., overlaying of concrete decks or slabs, jacketing of beam or columns, and filing material for precast concrete segments) [4,91,92,93,94]. In this case, heat treatment is not practically applied. The use of densified SF can be more essential for this type. This is because, in general, powder materials are continuously input and mixed into a mixer at an outdoor construction site; in this environment, the use of undensified SF can further deteriorate the limitations such as workplace dust generation, transportation, and the storage of raw materials. Thus, to fully examine the practical feasibility of densified SF, it is also necessary to conduct an investigation of UHPFRC cured without heat treatment. Moreover, when heat treatment is not applied, the mechanical properties of UHPFRC can change more sensitively depending on the type of SF, because, as mentioned earlier, the physical role (i.e., micro filling effect) rather than the chemical role (i.e., the pozzolanic reaction) can significantly contribute to the properties. In this case, a decrease in concrete performance due to a decrease in the dispersion efficiency of SF can be more pronounced.

**Figure 9:**
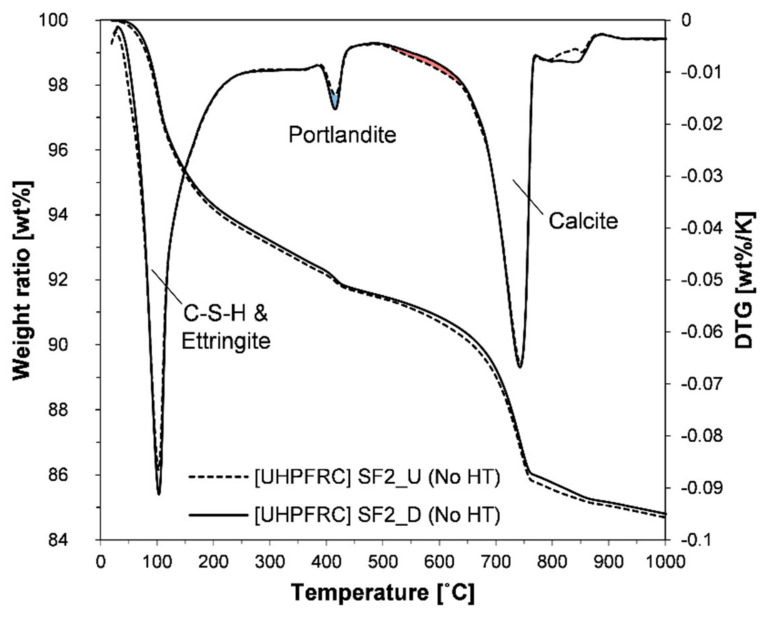
TG and DTG curves of ambient-cured paste samples at 28 days.

Figure 9 shows the results of TG analysis of the paste samples cured under ambient conditions for 28 days. To exclude factors other than the densification process, paste samples containing SF products from the same company were compared. Analyses of the results verify that the type of SF classified by this process does not affect the hydration reaction of UHPFRC, regardless of heat treatment. However, unlike results from the heat-treated samples (Figure 7b), a sharp peak was formed around 105 °C. This clearly confirms the presence of ettringite. The integration result of this sharp peak area was 5.76% and 5.75% in the SF2_U and SF2_D samples, respectively, confirming that there is no difference in the main hydration products (C-S-H and ettringite). This also indicates the possibility that there was no difference in the chemical reaction related to the formation of portlandite. Moreover, the result that no protrusion or peak was formed between 150 and 200 °C on the DTG curve shows that it is extremely difficult to form a new phase (hemi or mono carboaluminate) by direct reaction of calcite in the UHPFRC condition, regardless of the heat treatment. This is consistent with the results of previous studies [15,16,17].

When observing the peak at 400 °C on the DTG curve, there was a difference in the sizes of portlandite peaks between the two samples. The quantitative analysis results are presented in Table 4. The contents in the table were determined according to the tangential method along with the normalization method based on the weight at 550 °C [58]. The portlandite content of the sample with densified SF was 1.1 wt %, which was 0.21 wt % higher than the other sample of 0.89 wt %. However, based on the weight ratio of calcium oxide, most of the differences in the portlandite content are attributed to the formation of calcite (difference in CaO, _Portlandite_ = 0.16; difference in CaO, _calcite_ = 0.12). This also confirms that there was almost no difference between the two samples in the degree of pozzolanic reaction. The contribution of carbonation to the differences in portlandite content can also be visually shown in the blue and red shaded areas on the DTG curve. Since there was no difference between the two SF samples in the chemical reaction, the difference in the physical role (i.e., micro filling effect) caused by the dispersion efficiency can be considered as another potential factor affecting the mechanical properties. However, there was also no difference between the two samples in the compressive strength of UHPFRC at 7 and 28 days (Table 4). This demonstrates that the densified SF does not have any negative effect on either the physical or chemical roles of SF in the concrete, compared to the undensified SF.

SF products are only manufactured in limited regions of the world. Therefore, the products are inevitably transported and stored for a long time in a stacked state. Compared to undensified products, densified products are much freer from further agglomeration and quality changes after being manufactured [20]. The agglomeration by the densification process is reversible so that agglomerated SF particles can be effectively dispersed by an optimized mixing method, which is practically the only way this can be achieved. Along with this, the dispersion efficiency also depends on the chemical admixture used (performance or content), mixture composition, and water-to-powder ratio [37]. In particular, when a mixture of high volume powders is lumped and stiff like flour dough and a high mixing energy is applied to the mixture by a suitable mixer (e.g., twin shaft, planetary, or intensive mixer, etc.), the SF agglomerate can be effectively broken; eventually, their dispersion efficiency can be greatly increased [29,60]. In addition, the inclusion of PCE-based superplasticizer under these conditions and the application of high-speed mixing makes the homogeneous dispersion of powders including SF more effective. The composition of UHPFRC is characterized by a very low water-to-powder ratio and a very high content of PCE-based superplasticizer; this causes the dry materials to clump together in the mixing process. This is a clearly unique feature of UHPFRC that differs from other types of cement-based materials, and thus it can provide conditions for efficiently dispersing densified SF (equivalent to undensified products in terms of dispersion efficiency). Indeed, as all the results of this study consistently indicated, there was no notable difference between densified and undensified products in terms of the material properties of UHPFRC. Therefore, densified SF can be used to manufacture UHPFRC. In other words, although using this commercially optimal type of product, a compressive strength of >200 MPa can be achieved within 28 days, without disadvantages in workability and tensile properties.

## 4. Conclusions

This study was undertaken with the hypothesis that the composition and mixing method of UHPFRC are both unique and thus this condition is effective for dispersing the reversibly aggregated particles in densified SF. The UHPFRC is characterized by very high content in powders, PCE-based superplasticizer, and short steel fibers. Furthermore, a high-speed mixing process is included under all these conditions. In particular, since SF affects all important chemical and physical properties of the concrete (such as microstructure, hydration reaction, self-compacting ability, tensile and compressive strengths, and durability), the difference in dispersion degree should greatly affect the performance of the concrete. Experimental results on the comparison of undensified and densified SF demonstrated the validity of our hypothesis:

- Visual inspection and SEM image analysis confirmed that SF is composed of spherical nanoparticles, but, regardless of the type of SF product, they existed in the form of agglomerated lumps and the sizes of large ones reached several millimeters. The particle size analysis based on SEM images formed the size distribution in a smaller range compared to the results obtained by the DLS technique. The difference between the two techniques was attributed to the link of nanoparticles at a high temperature, the densification process or the agglomeration of nanoparticles thereafter, or the difference in dispersion efficiency during the ultrasonic treatment.
- The material properties of UHPFRC with densified and undensified SF were compared (their conditions other than the densification process were the same). Experimental results showed that there was no significant difference in workability, compressive strength, or flexural tensile strength between the two samples. Analysis of the hydration reaction based on XRD and TG also showed that there was almost no difference between the two samples in the formation or consumption of the main hydration products.
- When the samples were heat treated at 90 °C, portlandite was not identified because the chemical reaction related to the formation of this crystal was accelerated. This means that the pozzolanic reaction, which decisively affects the long-term strength and durability of the concrete, can be terminated significantly early due to the influence of limestone powder contained instead of Portland cement. Therefore, even when densified SF is used under standard heat treatment conditions, UHPFRC’s very high ultimate compressive strength (>200 MPa) can be ensured before 28 days.
- The results that the densified and undensified SFs did not differ in the hydration reaction and mechanical properties were also valid under air-dried curing conditions, without heat treatment. Thus, it was concluded that densified SF can be used for both precast and field casting UHPFRCs.

## Figures and Tables

**Table 1:** Chemical composition of silica fume samples.

Sample	SiO_2_	K_2_O	Al_2_O_3_	MgO	CaO	Na_2_O	SO_3_	Fe_2_O_3_	Others ^1^	LOI ^2^	Total
SF1_U	96.00	0.83	0.72	0.40	0.27	0.26	0.19	0.10	0.20	1.00	99.98
SF2_U	96.88	0.36	-	0.17	0.16	-	0.46	0.53	0.09	1.36	100.00
SF2_D	96.29	0.40	0.31	0.22	0.13	-	0.40	0.71	0.13	1.40	99.99

^1^ Components with less than 0.1% of content (Cl, P_2_O_5_, ZnO, MnO, CuO, Ru, Pd); ^2^ Loss on ignition.

**Table 2:** Mix proportions of UHPFRC (by weight of cement).

Cement	Silica Fume	Quartz Powder	Quartz Sand	Water	Superplasticizer ^1^	Steel Fiber ^2^
1	0.25	0.35	1.1	0.25	0.0012	2%

^1^ Solid contents; ^2^ By volume of UHPFRC.

**Table 3:** Average particle size and specific surface area of SF samples.

Sample Name	SF1_U	SF2_U	SF2_D
Average particle size by DLS (nm)	440	300	311
Specific surface area by BET method (m^2^/g)	23.8	25.3	22.9

**Table 4:** Portlandite and calcite contents and compressive strength of ambient-cured UHPFRC.

Sample	Quantitative Analysis by TG (wt %)				Compressive Strength (MPa)	
	Portlandite	CaO, _Portlandite_	Calcite	CaO, _Calcite_	7 Days	28 Days
[UHPFRC] SF2_U (No HT)	0.89	0.67	10.12	5.67	117.01 ± 1.38	154.01 ± 5.12
[UHPFRC] SF2_D (No HT)	1.10	0.83	9.92	5.55	117.17 ± 2.55	154.56 ± 4.02

## References

[1] Richard P., Cheyrezy M. (1995). Composition of reactive powder concretes. Cem. Concr. Res..

[2] De Larrard F., Sedran T. (1994). Optimization of ultra-high-performance concrete by the use of a packing model. Cem. Concr. Res..

[3] Brühwiler E., Denarié E. Rehabilitation of concrete structures using ultra-high performance fibre reinforced concrete. Proceedings of the Second International Symposium on Ultra High Performance Concrete.

[4] Bastien-Masse M., Brühwiler E. (2016). Experimental investigation on punching resistance of R-UHPFRC–RC composite slabs. Mater. Struct..

[5] Schmidt M., Fehling E. (2005). Ultra-high-performance concrete: Research, development and application in Europe. ACI Spec. Publ..

[6] Wille A.E.N.K., Gustavo J.P.-M. (2011). Ultra-high performance concrete with compressive strength exceeding 150 MPa (22 ksi): A simpler way. ACI Mater. J..

[7] Fontana P., Lehmann C., Müller U., Meng B. Reactivity of mineral additions in autoclaved UHPC. Proceedings of the International RILEM Conference on Material Science.

[8] Huang W., Kazemi-Kamyab H., Sun W., Scrivener K. (2017). Effect of replacement of silica fume with calcined clay on the hydration and microstructural development of eco-UHPFRC. Mater. Des..

[9] Hafiz M.A., Skibsted J., Denarié E. (2020). Influence of low curing temperatures on the tensile response of low clinker strain hardening UHPFRC under full restraint. Cem. Concr. Res..

[10] Ibrahim M.A., Farhat M., Issa M.A., Hasse J.A. (2017). Effect of material constituents on mechanical and fracture mechanics properties of ultra-high-performance concrete. ACI Mater. J..

[11] Zhang X., Zhao S., Liu Z., Wang F. (2019). Utilization of steel slag in ultra-high performance concrete with enhanced eco-friendliness. Constr. Build. Mater..

[12] Norhasri M.S.M., Hamidah M.S., Fadzil A.M. (2019). Inclusion of nano metaclayed as additive in ultra high performance concrete (UHPC). Constr. Build. Mater..

[13] Mo Z., Wang R., Gao X. (2020). Hydration and mechanical properties of UHPC matrix containing limestone and different levels of metakaolin. Constr. Build. Mater..

[14] Yu R., Spiesz P., Brouwers H.J.H. (2015). Development of an eco-friendly Ultra-High Performance Concrete (UHPC) with efficient cement and mineral admixtures uses. Cem. Concr. Compos..

[15] Huang W., Kazemi-Kamyab H., Sun W., Scrivener K. (2017). Effect of cement substitution by limestone on the hydration and microstructural development of ultra-high performance concrete (UHPC). Cem. Concr. Compos..

[16] Kang S.-H., Jeong Y., Tan K.H., Moon J. (2018). The use of limestone to replace physical filler of quartz powder in UHPFRC. Cem. Concr. Compos..

[17] Kang S.-H., Jeong Y., Tan K.H., Moon J. (2019). High-volume use of limestone in ultra-high performance fiber-reinforced concrete for reducing cement content and autogenous shrinkage. Constr. Build. Mater..

[18] Li P.P., Brouwers H.J.H., Chen W., Yu Q.L. (2020). Optimization and characterization of high-volume limestone powder in sustainable ultra-high performance concrete. Constr. Build. Mater..

[19] Li P.P., Yu Q.L., Brouwers H.J.H., Chen W. (2019). Conceptual design and performance evaluation of two-stage ultra-low binder ultra-high performance concrete. Cem. Concr. Res..

[20] ACI Committee 234 (2000). Guide for the Use of Silica Fume in Concrete.

[21] Ahmad S., Mohaisen K.O., Adekunle S.K., Al-Dulaijan S.U., Maslehuddin M. (2019). Influence of admixing natural pozzolan as partial replacement of cement and microsilica in UHPC mixtures. Constr. Build. Mater..

[22] De Larrard F. (1989). Ultrafine particles for the making of very high strength concretes. Cem. Concr. Res..

[23] Fehling E., Schmidt M., Walraven J., Leutbecher T., Fröhlich S. (2014). Ultra-High Performance Concrete UHPC: Fundamentals, Design, Examples.

[24] Kang S.-H., Hong S.-G., Moon J. (2019). The use of rice husk ash as reactive filler in ultra-high performance concrete. Cem. Concr. Res..

[25] Oertel T., Hutter F., Tänzer R., Helbig U., Sextl G. (2013). Primary particle size and agglomerate size effects of amorphous silica in ultra-high performance concrete. Cem. Concr. Compos..

[26] Loukili A., Khelidj A., Richard P. (1999). Hydration kinetics, change of relative humidity, and autogenous shrinkage of ultra-high-strength concrete. Cem. Concr. Res..

[27] Siddique R., Khan M.I. (2011). Supplementary Cementing Materials.

[28] Chan Y.-W., Chu S.-H. (2004). Effect of silica fume on steel fiber bond characteristics in reactive powder concrete. Cem. Concr. Res..

[29] Holland T.C. (2005). Silica Fume User’s Manual.

[30] Lothenbach B., Scrivener K., Hooton R.D. (2011). Supplementary cementitious materials. Cem. Concr. Res..

[31] Pfeifer C., Moeser B., Weber C., Stark J. Investigations of the pozzolanic reaction of silica fume in Ultra-high performance concrete (UHPC). Proceedings of the International RILEM Conference on Material Science-MATSCI.

[32] Oertel T., Hutter F., Helbig U., Sextl G. (2014). Amorphous silica in ultra-high performance concrete: First hour of hydration. Cem. Concr. Res..

[33] Van Tuan N., Ye G., van Breugel K., Copuroglu O. (2011). Hydration and microstructure of ultra high performance concrete incorporating rice husk ash. Cem. Concr. Res..

[34] St John D.A. (1994). The Dispersion of Silica Fume.

[35] Scrivener K., Young J.F. (1997). Mechanisms of Chemical Degradation of Cement-Based Systems.

[36] Pedro D., de Brito J., Evangelista L. (2017). Evaluation of high-performance concrete with recycled aggregates: Use of densified silica fume as cement replacement. Constr. Build. Mater..

[37] Bapat J.D. (2012). Mineral Admixtures in Cement and Concrete.

[38] Adil G., Kevern J.T., Mann D. (2020). Influence of silica fume on mechanical and durability of pervious concrete. Constr. Build. Mater..

[39] St John D.A., McLeod L.C., Milestone N.B. (1993). An Investigation of the Mixing and Properties of DSP Mortars Made from New Zealand Cements and Aggregates.

[40] Diamond S., Sahu S., Thaulow N. (2004). Reaction products of densified silica fume agglomerates in concrete. Cem. Concr. Res..

[41] Diamond S., Sahu S. (2006). Densified silica fume: Particle sizes and dispersion in concrete. Mater. Struct..

[42] Kolderup H. (1977). Particle size distribution of fumes formed by ferrosilicon production. J. Air Pollut. Control. Assoc..

[43] Möser B., Pfeifer C. Microstructure and durability of ultra-high performance concrete. Proceedings of the Second International Symposium on Ultra High Performance Concrete.

[44] Wang X., Huang J., Dai S., Ma B., Tan H., Jiang Q. (2019). Effect of silica fume particle dispersion and distribution on the performance of cementitious materials: A theoretical analysis of optimal sonication treatment time. Constr. Build. Mater..

[45] Arvaniti E.C., Juenger M.C.G., Bernal S.A., Duchesne J., Courard L., Leroy S., Provis J.L., Klemm A., De Belie N. (2015). Determination of particle size, surface area, and shape of supplementary cementitious materials by different techniques. Mater. Struct..

[46] Rodríguez E.D., Soriano L., Payá J., Borrachero M.V., Monzó J.M. (2012). Increase of the reactivity of densified silica fume by sonication treatment. Ultrason. Sonochem..

[47] Ma R., Guo L., Sun W., Rong Z. (2018). Well-dispersed silica fume by surface modification and the control of cement hydration. Adv. Civ. Eng..

[48] Bianchi G.Q. (2014). Application of Nano-Silica in Concrete. Ph.D. Thesis.

[49] Foerter-Barth U., Teipel U., Massacci P. (2000). Characterization of particles by means of laser light diffraction and dynamic light scattering. Developments in Mineral Processing.

[50] Malm A.V., Corbett J.C.W. (2019). Improved dynamic light scattering using an adaptive and statistically driven time resolved treatment of correlation data. Sci. Rep..

[51] Land G., Stephan D. (2012). The influence of nano-silica on the hydration of ordinary Portland cement. J. Mater. Sci..

[52] Lowke D., Schiessl P. Effect of mixing energy on fresh properties of SCC. Proceedings of the 4th International RILEM Symposium on Self-Compacting Concrete.

[53] Dils J., De Schutter G., Boel V. (2012). Influence of mixing procedure and mixer type on fresh and hardened properties of concrete: A review. Mater. Struct..

[54] (2014). ISO 13322-1:2014. Particle Size Analysis—Image Analysis Methods—Part 1: Static image analysis methods.

[55] (2014). ASTM C230/C230M-14. Standard Specification for Flow Table for Use in Tests of Hydraulic Cement.

[56] (2016). ASTM C109/C109M-16a. Standard Test Method for Compressive Strength of Hydraulic Cement Mortars (Using 2-in. or [50-mm] Cube Specimens).

[57] (2009). ISO 679. Cement–Test Methods–Determination of Strength.

[58] Scrivener K., Snellings R., Lothenbach B. (2016). A Practical Guide to Microstructural Analysis of Cementitious Materials.

[59] Snellings R., Chwast J., Cizer Ö., De Belie N., Dhandapani Y., Durdzinski P., Elsen J., Haufe J., Hooton D., Patapy C. (2018). Report of TC 238-SCM: Hydration stoppage methods for phase assemblage studies of blended cements—Results of a round robin test. Mater. Struct..

[60] Hewlett P. (2010). Lea’s Chemistry of Cement and Concrete.

[61] Kaasalainen M., Aseyev V., von Haartman E., Karaman D.Ş., Mäkilä E., Tenhu H., Rosenholm J., Salonen J. (2017). Size, stability, and porosity of mesoporous nanoparticles characterized with light scattering. Nanoscale Res. Lett..

[62] Snellings R., Mertens G., Elsen J. (2012). Supplementary Cementitious Materials. Rev. Mineral. Geochem..

[63] (2016). NF P18-470. Ultra-High Performance Fibre-Reinforced Concrete—Specifications, Performance, Production and Conformity.

[64] Sobolev K., Flores I., Hermosillo R., Torres-Martínez L.M. Nanomaterials and nanotechnology for high-performance cement composites. Proceedings of the ACI Session on Nanotechnology of Concrete: Recent Developments and Future Perspectives.

[65] Deshini A., Ioannides A.M. (2012). Undispersed agglomerates and the strength of microsilica concrete. Int. J. Pavement Eng..

[66] Lagerblad B., Utkin P. (1993). Silica Granulates in Concrete: Dispersion and Durability Aspects.

[67] Marusin S.L., Shotwell L.B. (2000). Alkali-silica reaction in concrete caused by densified silica fume lumps: A case study. Cem. Concr. Aggreg..

[68] Shayan A., Quick G.W., Lancucki C.J. (1993). Morphological, mineralogical and chemical features of steam-cured concretes containing densified silica fume and various alkali levels. Adv. Cem. Res..

[69] Baweja T.C.D., Bucea L. (2003). Investigation of Dispersion Levels of Silica Fume in Pastes, Mortars, and Concrete.

[70] Cao Y., Yu Q.L., Brouwers H.J.H., Chen W. (2019). Predicting the rate effects on hooked-end fiber pullout performance from Ultra-High Performance Concrete (UHPC). Cem. Concr. Res..

[71] Hafiz M.A., Denarié E. (2020). Tensile response of UHPFRC under very low strain rates and low temperatures. Cem. Concr. Res..

[72] Kang S.-H., Lee J.-H., Hong S.-G., Moon J. (2017). Microstructural investigation of heat-treated ultra-high performance concrete for optimum production. Materials.

[73] Zhou B., Uchida Y. (2017). Influence of flowability, casting time and formwork geometry on fiber orientation and mechanical properties of UHPFRC. Cem. Concr. Res..

[74] Abrishambaf A., Pimentel M., Nunes S. (2017). Influence of fibre orientation on the tensile behaviour of ultra-high performance fibre reinforced cementitious composites. Cem. Concr. Res..

[75] Huang H., Gao X., Li Y., Su A. (2020). SPH simulation and experimental investigation of fiber orientation in UHPC beams with different placements. Constr. Build. Mater..

[76] Larsen I.L., Thorstensen R.T. (2020). The influence of steel fibres on compressive and tensile strength of ultra high performance concrete: A review. Constr. Build. Mater..

[77] Taylor H., Famy C., Scrivener K. (2001). Delayed ettringite formation. Cem. Concr. Res..

[78] Heinz D., Urbonas L., Gerlicher T. Effect of heat treatment method on the properties of UHPC. Proceedings of the 3rd International Symposium on Ultra High Performance Concrete and Nanotechnology for High Performance Construction Materials.

[79] Jensen O.M., Hansen P.F. (2001). Water-entrained cement-based materials: I. Principles and theoretical background. Cem. Concr. Res..

[80] Justs J., Wyrzykowski M., Bajare D., Lura P. (2015). Internal curing by superabsorbent polymers in ultra-high performance concrete. Cem. Concr. Res..

[81] Heinz D., Ludwig H.-M. Heat treatment and the risk of DEF delayed ettringite formation in UHPC. Proceedings of the 1st International Symposium on Ultra-High Performance Concrete.

[82] Selleng C., Meng B., Fontana P. Phase composition and strength of thermally treated UHPC. Proceedings of the 4th International Symposium on Ultra-High Performance Concrete and High Performance Materials.

[83] Selleng C., Fontana P., Meng B. Possibilities for improving the properties of UHPC by means of thermal treatment. Proceedings of the AFGC-ACI-fib-RILEM PRO 106: Ultra-High Performance Fibre-Reinforced Concrete (UHPFRC 2017).

[84] Korpa A., Kowald T., Trettin R. (2009). Phase development in normal and ultra high performance cementitious systems by quantitative X-ray analysis and thermoanalytical methods. Cem. Concr. Res..

[85] Cheyrezy M., Maret V., Frouin L. (1995). Microstructural analysis of RPC (Reactive Powder Concrete). Cem. Concr. Res..

[86] Jensen O.M., Hansen P.F. (1999). Influence of temperature on autogenous deformation and relative humidity change in hardening cement paste. Cem. Concr. Res..

[87] Alexander G.B., Heston W.M., Iler R.K. (1954). The solubility of amorphous silica in water. J. Phys. Chem..

[88] Zhang M.-H., Gjørv O.E. (1991). Effect of silica fume on cement hydration in low porosity cement pastes. Cem. Concr. Res..

[89] Schachinger I., Hilbig H., Stengel T. Effect of curing temperature at an early age on the long-term strength development of UHPC. Proceedings of the Second International Symposium on Ultra High Performance Concrete.

[90] Chung D.D.L. (2002). Review: Improving cement-based materials by using silica fume. J. Mater. Sci..

[91] Talayeh N., Eugen B. (2013). Experimental investigation on reinforced ultra-high-performance fiber-reinforced concrete composite beams subjected to combined bending and shear. ACI Struct. J..

[92] Lampropoulos A.P., Paschalis S.A., Tsioulou O.T., Dritsos S.E. (2016). Strengthening of reinforced concrete beams using ultra high performance fibre reinforced concrete (UHPFRC). Eng. Struct..

[93] Ford E.L., Hoover C.G., Mobasher B., Neithalath N. (2020). Relating the nano-mechanical response and qualitative chemical maps of multi-component ultra-high performance cementitious binders. Constr. Build. Mater..

[94] Ali Dadvar S., Mostofinejad D., Bahmani H. (2020). Strengthening of RC columns by ultra-high performance fiber reinforced concrete (UHPFRC) jacketing. Constr. Build. Mater..

